# Microcosting Study of Genomic Profiling for Precision Cancer Medicine: Application from the National Infrastructure for Precision Diagnostics in Norway

**DOI:** 10.1016/j.jmoldx.2025.06.006

**Published:** 2025-07-23

**Authors:** Pia S. Henkel, Eline Aas, Hege G. Russnes, Ingrid Dyvik, G. Live Fagereng, Åslaug Helland, Hanna Røgenes, Tonje G. Lien, Kine Pedersen

**Affiliations:** ∗ Institute of Health and Society, University of Oslo, Oslo, Norway; † Division for Health Services, Norwegian Institute of Public Health, Oslo, Norway; ‡ Department of Pathology, Division of Laboratory Medicine, Oslo University Hospital, Oslo, Norway; § Institute for Cancer Research, Oslo University Hospital, Oslo, Norway; ¶ Institute of Clinical Medicine, University of Oslo, Oslo, Norway

## Abstract

Detailed cost analyses of genomic profiling for precision cancer medicine can inform strategic planning and cost-effectiveness analysis. A flexible costing framework was developed in this study to conduct microcosting of genomic profiling in precision cancer medicine using the broad gene panel TruSight Oncology 500 and accounting for its integration into the molecular tumor board within the national Infrastructure for Precision Diagnostics in Norway. The framework enables calculation of costs per sample, by workflow steps and cost categories. Site visits and discussions with staff at Oslo University Hospital informed the diagnostic workflow, validation of the framework, and resource use inputs. Sensitivity analysis addressed alternative resource use estimates, higher batch sizes, and investment costs for automation of the library preparation step. Total costs per sample were $2944 USD, ranging from $2366 to $4307 when considering uncertainties in estimates. Consumables and personnel were the most resource-intensive cost categories across analyses. Automating the resource-intensive library preparation step enabled a higher weekly batch size with slightly lower costs per sample ($2881) despite the additional equipment costs. The dynamic costing framework highlights how the choice of equipment and batch sizes affects sample costs and personnel needs for genomic profiling. Consumables and personnel offer the largest potential for costs savings, but potential personnel bottlenecks need to be considered when further upscaling capacity.

Advancements in precision cancer medicine rely on advanced diagnostic methods to test patients with cancer for biomarkers that predict response and resistance to targeted therapies. As an increasing number of targetable alterations are identified and validated, genomic profiling has emerged as a key diagnostic tool to inform therapy selection in clinical practice.^1^^,^^2^ Broad next-generation sequencing (NGS) panels enable high-throughput analysis of genomic alterations across hundreds of genes simultaneously.^1^ Despite decreasing costs of sequencing in general,^3^ broad gene panel testing represents a more complex diagnostic workflow and remains costly compared with single-gene tests or smaller gene panels.^4^ Cost concerns and insufficient reimbursement are among of the main reasons for impeded or limited implementation of genomic profiling in routine clinical practice.^4^, ^5^, ^6^

Estimating the costs of genomic profiling can provide valuable insight for several applications. First, cost estimates can be used for hospital budgeting and can inform the definition and valuation of reimbursement fees in clinical practice. In turn, this could support decisions on reimbursement of testing and implementation in the health care system. Mapping out the diagnostic workflow and identifying the necessary resources, such as personnel needs and instrumentation, can support the identification of bottlenecks for upscaling the sequencing capacity. This is particularly important for strategic planning of genomic profiling for larger populations. Finally, according to Norwegian priority setting criteria for health care services, evaluation of costs and health benefits is required before implementation.^7^ Thus, cost estimates are a necessary input for cost-effectiveness analyses evaluating the implementation of genomic profiling into diagnostic pathways for patients with cancer, as well as for the evaluation of precision cancer medicine treatments that require biomarker testing.

Because of the diverse landscape of sequencing platforms, genomic assays, and operational procedures in and outside of the laboratories, determining a standard microcosting approach for genomic profiling is challenging.^8^ Despite following a microcosting approach, previous costing studies of genomic sequencing for cancer care exhibit substantial differences in the level of detail used to report resource use per step (ie, a sequence of defined activities that form the diagnostic workflow).^8^ To facilitate comparisons across studies, some of these studies propose frameworks or a sequence of generalized steps to standardize the reporting of costs in genomic sequencing.^9^^,^^10^ Identifying cost-driving steps may help explain differences in total costs between studies and may improve the quality and consistency of measuring the cost of genomic sequencing, alongside precise technology definition, detailed and comprehensive reporting of resource use, and critical consideration of the context in which the analysis is performed.^11^

To support implementation of genomic profiling and associated decision-making processes, this study aimed to develop a microcosting framework and provide evidence on the resource use for genomic profiling within the application example of the national Infrastructure for Precision Diagnostics (InPreD) in Norway. The implementation of genomic profiling in InPreD aims to identify patients with advanced cancer who may benefit from targeted therapies, including experimental cancer therapy, highlighting the necessity of patient-centric variant interpretation and multidisciplinary discussion within a molecular tumor board. This study further aimed to enable flexible adaptation to other settings and identification of cost drivers at different batch sizes with a microcosting framework.

## Materials and Methods

### Analytic Setting

InPreD is a national infrastructure and implementation program for precision diagnostics in cancer care within the public health care system in Norway, involving all six university hospitals.^12^ The collaborative public program was launched in 2019 by the Norwegian Health Authorities and aims at developing interdisciplinary environments for advanced cancer diagnostics across the country. InPreD has a particular assignment to implement experimental diagnostic methods into public health care to facilitate access to clinical trials, including the national researcher-initiated precision cancer medicine trial Improving Public Cancer Care by Implementing Precision Medicine in Norway (IMPRESS-Norway).^13^ Patients from all hospitals in Norway with treatment-refractory advanced cancer can be referred to one of the four operative InPreD nodes for genomic profiling using the broad gene panel TruSight Oncology 500 (Illumina, San Diego, CA). Results are discussed at the weekly national molecular tumor board (MTB) to guide molecular-based therapy recommendations and assess the potential eligibility for experimental therapy in biomarker-defined clinical trials, early-access programs, or additional therapy as standard of care.^12^ As of November 2024, >2300 patient samples have been analyzed and discussed at the MTB.

Using a health care perspective, the costs per patient sample were estimated by identifying, measuring, and valuing cost components.^14^ A microcosting approach was used where possible, where each component is identified, measured, and valued separately using unit costs.^15^^,^^16^ In the Norwegian guidelines for single technology assessments for diagnostics,^17^ no costing method is explicitly recommended or required. However, as suggested in these guidelines, the Norwegian unit costs and market prices were used, and the capital costs for large medical equipment were calculated by amortizing over the equipment's lifetime and including maintenance costs wherever possible. Results will be presented in 2024 US dollars ($) using the 2024 currency exchange rate of $1 = 10.7433 NOK (Norwegian kroner, exchange rate obtained from Norges Bank, *https://www.norges-bank.no/en/topics/Statistics/exchange_rates/?tab=currency&id=USD&frequencyTab=3*, last accessed April 10, 2025). This study did not involve individual-level data collected from humans or biosamples collected from humans.

### Costing Framework

To develop a costing framework for genomic profiling and validate results by comparing cost estimates, previous costing studies of genomic sequencing were reviewed to identify common approaches for categorizing resource use. In addition to the seven studies^9^^,^^18^, ^19^, ^20^, ^21^, ^22^, ^23^ included in a systematic review,^8^ one additional and five newer costing studies were identified in an unstructured search.^10^^,^^24^, ^25^, ^26^, ^27^, ^28^ Across these 13 studies, there was considerable variation in the reported cost categories and steps in the diagnostic workflow.

**Figure 1:**
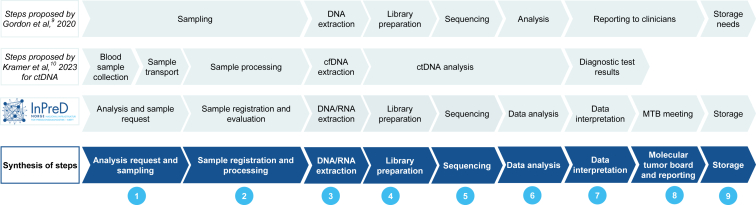
Steps identified in the diagnostic workflow for genomic profiling. This figure presents an overview of the proposed sequence of generalized activity steps to structure the reporting of costs for genomic profiling for precision cancer medicine. The first and second row illustrate the steps proposed in two previous costing studies, which, together with the third row, illustrating the steps in the Infrastructure for Precision Diagnostics (InPreD) workflow, were used to generate a synthesis of steps. The last row presents the synthesis of steps used in the study and is meant to be relevant for different types of analysis, such as circulating tumor DNA (ctDNA) analysis, as well. cfDNA, cell-free DNA; MTB, molecular tumor board.

Two of the previous studies propose a sequence of generalized steps (ie, the defined activities that form the diagnostic workflow) to standardize the reporting of costs.^9^^,^^10^ On the basis of these two studies and the diagnostic workflow in InPreD, the diagnostic workflow of genomic profiling was structured into nine steps (Figure 1). Step 1: Analysis request and sampling involves registration of the analysis request sent by the treating physician for their patient, selection and request of archival tumor tissue samples located at local hospitals (most often formalin-fixed, paraffin-embedded tissue), and the transportation of the samples to the regional InPreD laboratory. Step 2: Sample registration and processing includes the evaluation, selection, and registration of the selected tumor tissue sample by a pathologist, followed by sectioning and macrodissection of the tumor areas. Step 3: DNA/RNA extraction includes lysis and separation of DNA and RNA from the tumor sample, extraction and dilution of DNA and RNA, and quality control. Step 4: Library preparation includes DNA fragmentation, synthesis of cDNA from RNA, end repair and A-tailing, adapter ligation, index PCR, first and second hybridization, first and second capture, library amplification, quality control, and normalization. Step 5: Sequencing includes library pooling, dilution and denaturation, sequencing, and data transfer. Step 6: Data analysis includes primary analysis, in-house post-processing, quality control, and generation of an initial molecular report. Step 7: Data interpretation includes patient-centric variant interpretation by the molecular biologists. Step 8: Molecular tumor board and reporting involves the meeting time for a two-step MTB process, finalization of the molecular report, and the entry of the final pathology report into the pathology information system. Step 9: Storage relates to the deposition of genomic data after reporting of the results.

The most frequently used cost categories in existing microcosting studies were selected to further structure the costing framework, (ie, consumables, personnel, and equipment costs). Equipment costs included costs for the laboratory spaces, and software and storage costs were included further to emphasize the need for data infrastructure in the diagnostic workflow. Overhead costs for resource use that is not directly linked to an individual sample were included by a mark-up of 20% on the total costs (eg, costs for administrative departments, office supplies, rent for nonlaboratory spaces), based on internal guidelines at Oslo University Hospital. The costs per patient sample will be presented both per step and per category.

To allow cost estimates to vary with batch size and to display capacity constraints, costs were categorized as variable, fixed, or step-fixed costs.^14^^,^^29^ Variable costs are incurred per sample, and thus increase proportionally to the number of samples run in one workflow. Examples include single-use sample tubes or personnel time to discuss an individual patient case in the MTB. Fixed costs are incurred independent of the number of samples for a given time period (eg, purchase of large laboratory equipment or software licenses without capacity restrictions). Step-fixed costs are fixed costs within a certain activity level, and thus increase stepwise if the number of samples run (ie, the batch size) is higher than the constraint. One example for step-fixed costs is the personnel time required for certain work steps in the library preparation process that are optimized for up to a certain number of samples as per current laboratory protocols. A second example for step-fixed costs are consumables with a fixed capacity (eg, single-use tube-strips with eight wells or reagent kits for a specified number of samples to be used for the sequencing platform).

Total consumable and personnel costs per batch were calculated by multiplying the unit price or hourly wage per profession with the number of units or the working time in hours needed per batch, considering the batch size and whether resource use was variable or step fixed. Dividing the total costs per batch by the batch size resulted in the costs per sample. Equipment costs were calculated as annual capital costs considering the purchase price, an interest rate of 4% per year, the expected lifetime of the equipment, plus annual maintenance costs and divided by the yearly number of samples. Costs for the laboratory space were calculated on the basis of the square meter area multiplied with internal per square meter costs from hospital guidelines and divided by the yearly number of samples. A percentage weighting for cost allocation to other steps or use by other departments was incorporated. The software costs per sample were calculated as the weighted annual software license costs, again using a weight factor informed by internal experts for cost allocation, divided by the yearly number of samples. As part of software costs, costs for storage were calculated by multiplying the average data volume per patient sample with the storage costs per gigabyte on a server for sensitive data, assuming a storage time frame of 10 years.

### Data Input

To inform the identification, measurement, and valuation of cost components, several site visits to the InPreD node at Oslo University Hospital (OUS) were conducted between April and October 2024. Meetings were held with InPreD personnel at OUS (laboratory leaders and engineers, biostatisticians, molecular biologists, pathologist, and project coordinators). Notes and copies from these meetings, MTB meetings, and laboratory protocols were used to understand the diagnostic workflow, develop the costing framework in Excel version 2408 (Microsoft, Redmond, WA), and identify resource items. The list of identified resource items was validated by InPreD personnel, who also provided inputs for all parameters measuring resource use: number of units of consumables needed, working times per step for trained personnel, the number of and expected lifetime of the equipment, and resource use related to software and storage. To account for variation in working times across specific steps, a range consisting of a lower value, a higher value, and a best estimate for the average working time (referred to as base case) was agreed on in discussion with OUS personnel. Similarly, a lower and higher value was indicated for equipment lifetime and data storage volume.

Each item was valued using Norwegian data: union agreements from the Norwegian Medical Association and Norwegian Union of Municipal and General Employees [*https://www.legeforeningen.no/jus-og-arbeidsliv/avtaler-for/leger-ansatt-pa-sykehus/offentlige-sykehus/minimumslonn-sykehus* (Norwegian) and *https://www.fagforbundet.no/lonn-og-avtaler/spekter/sykehus* (Norwegian), last accessed April 10, 2025] plus 13% employer contributions and 25% social security costs (Norwegian Medical Products Agency, *https://www.dmp.no/en/public-funding-and-pricing/health-technology-assessments/medicines/submission-of-documentation-for-single-technology-assessment-of-pharmaceuticals/unit-cost-database*, last accessed April 10, 2025) for annual salary of trained staff, purchasing prices for consumables, equipment, and software from Norwegian or international online distributors or internal price information from OUS. If necessary, prices were converted using the 2024 exchange rate of $1 = 10.7433 NOK and €1 = 11.6276 NOK (Norges Bank, *https://www.norges-bank.no/en/topics/Statistics/exchange_rates/?tab=currency&id=EUR&frequencyTab=3*, last accessed April 10, 2025).

### Statistical Analysis

The batch size was defined as a flexible parameter in this study, with the base case being the capacity limit for one weekly run at InPreD OUS at the time of data collection (12 samples per week as of June 2024). Different batch sizes (1, 4, 8, 12, 16, 24, 32, and 64) were then simulated to explore costs drivers at different capacity levels. Costs resulting from automating the library preparation workflow (16 samples per week) were also analyzed, as implemented at InPreD OUS autumn 2024, and higher batch sizes were simulated with the automated workflow as well. To account for alternative scenarios in the valuation of unit costs (eg, because of potential bulk discounts in hospital purchasing agreements), the possibility to vary the unit costs with a percentage discount or surcharge was implemented. To further explore alternative costing assumptions, the possibility to weigh the total costs per step with a percentage value was included. This allows using a weight of >100% (eg, to account for the processing of control samples or repeated analysis in case of failure of a quality control step). In an additional scenario, investment costs for the development of code for the analysis and reporting of sequencing data were included, reflecting 16 months of work for one bioinformatician. These costs can be considered sunk costs and not part of the continuous practice but still represent an important investment cost.

### Availability of the Microcosting Framework

The microcosting framework, including resource use estimates and valuations, is available as Supplemental Appendix S1.

## Results

### Resulting Costs per Sample

In the base case analysis, based on 12 samples processed per week and using best estimates for personnel times and equipment lifetime, the total costs per patient sample amount to $2944 (Table 1). Consumables and personnel were the largest cost components, with 34% and 35% of total costs each. Within consumables, >80% of costs relate to the reagent kits for library preparation and sequencing, with typical rebate. The cost category accounting for the smallest proportion of total costs is software and data storage costs, which contributes to 1% of total costs. The most resource-intensive step is library preparation, with 34% of total costs, particularly due to high costs for consumables related to this step. Using the low values for working times and equipment lifetime, the cost per sample was 20% lower than the base case estimate, with $2366. Using the high values, on the other hand, would lead to 46% higher costs of $4307 (Supplemental Table S1). Varying these measurement parameters had no impact on the costs of consumables.

**Table 1:** Costs per Sample in Base Case Analysis for a Weekly Sample Size of 12

Step/cost category	Consumables, $	Personnel, $	Equipment, $	Software and storage, $	Overhead 20%, $	Total costs per sample, $	% Of total costs
1: Analysis request and sampling	0	150	0	0	30	179	6
2: Sample registration and processing	21	107	12	0	28	168	6
3: DNA/RNA extraction	33	94	26	0	31	183	6
4: Library preparation	688	101	56	0	169	1014	34
5: Sequencing	273	31	279	25	122	729	25
6: Data analysis	0	48	0	0	10	57	2
7: Data interpretation	0	292	0	3	59	353	12
8: Molecular tumor board and reporting	0	207	1	0	41	249	8
9: Storage	0	0	0	9	2	10	<1
Total costs	1015	1029	374	36	491	2944	100
% Of total costs	34	35	13	1	17	100	

### Cost Composition for Different Batch Sizes and Automated Library Preparation

**Figure 2:**
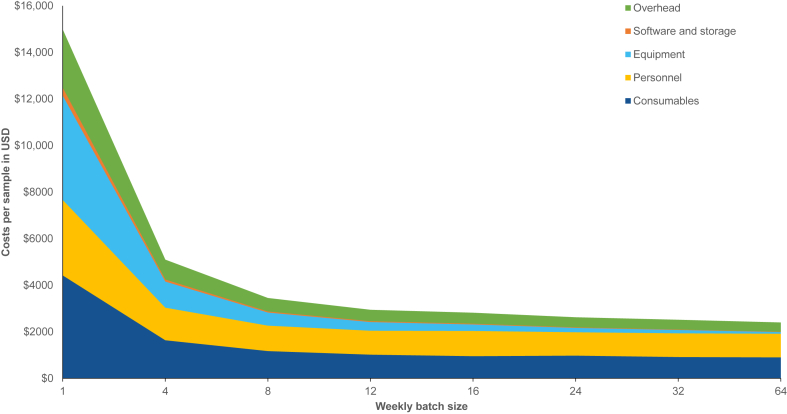
Costs per cost category along different batch sizes with manual library preparation. This stacked area diagram displays the development of the costs per sample along different weekly batch sizes, with the individual stacked areas representing the different cost categories. Best estimates for working time and equipment lifetime were used for the calculations.

When considering different batch sizes, the costs per sample are gradually decreasing with higher batch sizes (Figure 2). Equipment costs per sample continually decrease, whereas consumables and personnel costs, which typically reflect variable or step-fixed costs, only marginally reduce with increasing sample sizes.

**Figure 3:**
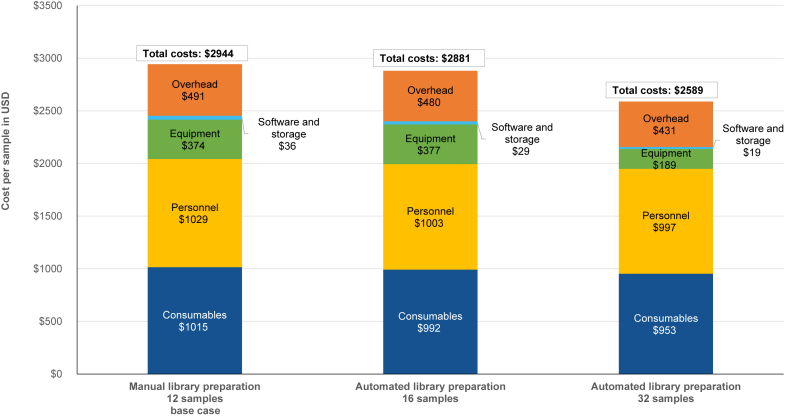
Cost per sample with manual compared with automated library preparation for different batch sizes. This stacked column chart displays the total cost per sample by the different cost categories for the base case costing analysis with manual library preparation of 12 weekly samples compared with automated library preparation of 16 and 32 weekly samples. Best estimates for working time and equipment lifetime were used for the calculations.

When considering higher batch sizes enabled by robotic automation of the library preparation step (ie, 16 weekly samples instead of 12), total costs per sample were $2881 and thus 2% lower (Figure 3). Because of capital costs for the laboratory robot, costs for equipment were slightly higher despite allocation of costs to more weekly samples. On the other hand, the reduction in laboratory engineer working time per sample resulted in lower personnel costs. Additionally, some cost components for consumables and software with fixed or step-fixed cost character contributed to lower costs per sample. When simulating 32 weekly samples, costs per sample would be $2589 with automated library preparation. However, processing ≥32 weekly samples would require additional staff, representing an important bottleneck.

### Scenario Analysis

If the prices of consumables were 50% lower, the total costs per sample would decrease by 21% (Table 2). As an example of the flexible analysis described in Figure 2, costs per sample would decrease by 11% when doubling the weekly sample size. Excluding the molecular tumor board and reporting (step 8) would result in total costs of $2695. When including the costs of bioinformatic development, the costs of software and storage increase from $36 to $135, resulting in a 4% cost increase in overall costs compared with the base case. For higher batch sizes, however, this inclusion of additional fixed costs would have almost no impact (Supplemental Figure S1). If a 10% cost increase is assumed for steps 3 to 5 (ie, DNA/RNA extraction, library preparation, and sequencing) to account for control samples and the possibility that samples fail the analysis, total costs increase by 7% to $3136 per sample.

**Table 2:** Cost per Sample in Scenario Analysis

Scenario	Consumables, $	Personnel, $	Equipment, $	Software and storage, $	Overhead 20%, $	Total costs per sample, $	% Change in costs
Base case	1015	1029	374	36	491	2944	Reference
50% Price reduction in consumables	507	1029	374	36	389	2335	–21
Doubling the weekly sample size to 24 samples	973	1003	187	22	437	2623	–11
Exclusion of step 8: molecular tumor board and reporting						2695	–8
Inclusion of bioinformatic development costs	1015	1029	374	135	510	3062	4
+10% for controls and sample failure in steps 3–5						3136	7

## Discussion

At current capacity levels, the cost per sample for genomic profiling using the Illumina TruSight Oncology 500 NGS assay at OUS are estimated at $2944 (low estimate, $2366; high estimate, $4307). The largest cost components are personnel with 35% and consumables with 34% of total costs, predominantly reagent kits for library preparation and sequencing. Higher batch sizes would lead to reductions in cost per sample, but consumables and molecular data interpretation effort with variable cost character would remain resource intensive.

### Strengths and Limitations

This study comprehensively assesses the costs related to genomic profiling in precision cancer medicine, including costs for patient-centric variant interpretation and integration into the MTB in a real-world setting. Costs are transparently reported and broken down in categories and steps, which enables the calculation of costs under different assumptions. For example, the costs of a certain step could be excluded and replaced with the service cost to a commercial provider if a part of the workflow is outsourced. Uncertainties in resource measurements and valuation are acknowledged in scenario analysis. The flexible framework allowed us to simulate costs at different test capacities for both manual and automated library preparation, which is important for strategic planning.

The decentralized approach of the InPreD program with several testing nodes may limit comparability with countries following a centralized approach or using commercial providers with high sample turnover. Despite being part of the public health care system, InPreD operates in the intersection between health care service and research, functioning primarily as a diagnostic platform for experimental therapy in precision cancer medicine. For example, the time spent on interpretation and MTB discussions includes comprehensive evaluation of each patient case with extensive manual work and literature searches to identify available evidence on each potential biomarker-diagnose-drug combination. Furthermore, the time spent in the national alignment meeting for molecular biologists, which is directly related to data interpretation, was included. However, other developing work was not accounted for, such as non-OUS personnel participating in the national MTB meetings as an interdisciplinary platform for alignment and competence building, nor costs related to testing and validation of new equipment, such as the laboratory robot. Including these costs would further increase total costs per sample and might be worthwhile to explore when the scope of the costing study goes beyond the current diagnostic pathways.

Approximating indirect costs and especially costs for spaces other than the laboratory (for instance, space for bioinformaticians and molecular biologists) by a 20% mark-up might not reflect the full use of resources and is a relevant limitation of this costing framework.

### Comparison with Previous Costing Studies

In the identified microcosting studies of genomic sequencing, costs are estimated for a variety of different applications, both for rare diseases and cancer care, and a multitude of testing technologies including smaller gene panels, broad gene panels, and whole-genome sequencing. This variety complicates comparisons on the cost level and analyzing trends over time. Therefore, comparisons were focused on cost estimates specifically for gene panel tests in cancer care. The Australian study that served as a reference for breaking down costs into steps found that consumables for library preparation and sequencing contributed to >50% of costs.^9^ The second study serving as reference for breaking down costs into steps also reported costs depending on the number of samples per week for a custom NGS panel covering 45 genes.^10^ A 2022 study on NGS panels used in non–small-cell lung cancer reported costs of 1778 Canadian dollars ($2187 inflated from 2019 to 2024) for a gene tumor panel covering 170 genes, with library preparation and sequencing being the main cost drivers.^25^ In a systematic review on the costs of NGS in oncology, costs for large gene panels were informed by three studies and ranged from $4011 to $8624 in 2023 USD,^30^ although none of these classified as a microcosting study.

The results of this study are in line with most studies reporting consumables as the most costly category (for instance, also in a study from France^26^ or the United Kingdom^22^). In contrast, this study finds a higher proportion of personnel costs (35%) than some of the previous studies. This is likely because of comprehensive inclusion of personnel activities, especially accounting for labor cost related to the MTB discussion and reporting, which reflects the rationale of InPreD to identify patients eligible for precision cancer medicine trials and accounts for 8% of total costs (step 8). Few of the other studies included software costs^20^^,^^21^; however, other studies included costs specifically related to bioinformatics,^19^^,^^24^^,^^28^ which presumably account for software and storage costs in addition to bioinformatic labor.

### Study Relevance and Need for Further Research

Using a flexible costing framework, this study showed that increasing capacities does to a certain extent reduce costs, which may support centralization of testing. However, costs are largely driven by variable costs for consumables, where economies of scale will only impact if hospitals are able to negotiate bulk discounts for consumables when processing many samples per year. Single-use laboratory consumables are also resource intensive from an environmental perspective, a topic increasingly gaining attention.^31^^,^^32^ If the environmental impacts of consumables become mandatory for inclusion in cost-effectiveness analysis informing priority setting,^33^ it is assumed that the consequences for genomic profiling, as well as other diagnostic tests, may be substantial. Furthermore, how much a cost category drives total costs does not necessarily indicate bottlenecks in the diagnostic workflow. For instance, although sample preparation might not be as costly as library preparation and data interpretation, evaluating and preparing the tumor samples for sequencing depends on the availability of qualified personnel. By implementing the cost type of step-fixed costs, a focus on capacity constraints was enabled in both the analysis and in the discussion with associated staff. Furthermore, higher batch sizes might require re-organization of the diagnostic workflow, training and alignment of additional staff, increased coordination effort, and additional space. Hence, extrapolating this costing analysis to high capacities might not be reasonable. In particular, inclusion of rental costs is not detailed and comprehensive enough to allow for the framework to be used for strategic planning of space.

Although potential costs of failure were explored in one of the scenarios, the true costs per patient sample will ultimately depend on the quality of the sample, the need for repeated analysis or new biopsy collection, and the effort needed to interpret and report on the genomic data generated through sequencing. In the future, this effort might be reduced by clinical decision support tools that could support the data interpretation effort of the molecular biologists, or automated processes for report generation. Besides the impact on costs, the quality of the sample also impacts specificity and sensitivity of the genomic testing, which should be explored for cost-effectiveness analysis and calls for further research.^34^ For a cost-effectiveness analysis, the average costs per patient sample for genomic profiling as calculated in this study need to be linked to the molecularly guided treatment recommendations in the molecular tumor board and the potential impact on survival and health-related quality of life from these recommendations. Hence, to facilitate cost-effectiveness analyses for genomic profiling, further research should focus on the clinical value of findings from genomic profiling and extend to the patient benefits and costs of subsequent treatment.

## Conclusion

This study provides a comprehensive estimation of the costs of consumables, personnel, equipment, software and storage, and overhead costs for genomic profiling within InPreD at OUS at current capacity level. Higher batch sizes, potentially enabled by robotic automation, would lead to reductions in costs per sample. However, consumables and personnel especially for data interpretation and reporting would remain resource intensive and offer the largest potential for further cost savings. To further upscale capacity, health care systems also need to consider potential personnel bottlenecks.

## Disclosure Statement

I.D., H.G.R., Å.H., and G.L.F. report receipt of research funding to the clinical trial IMPRESS-Norway for circulating tumor DNA tests from Illumina and Roche, but have not received any personal financial benefits or compensations related to this or other related research projects.

## Supplemental Data

Supplemental Appendix S1

Supplemental Figure 1 Costs per sample in scenario analyses along different batch sizes. This line chart displays the costs per sample along different batch sizes for the base case calculation using best estimates, low value and high value estimates for working time and equipment lifetime, and for the calculations in scenario analysis.
