# Supplementary figures and images for: Microcosting Study of Genomic Profiling for Precision Cancer Medicine: Application from the National Infrastructure for Precision Diagnostics in Norway
Source: J Mol Diagn. 2025 Jul 23;27(10):945–53. doi: 10.1016/j.jmoldx.2025.06.006 (PMC12597535; doi:10.1016/j.jmoldx.2025.06.006)

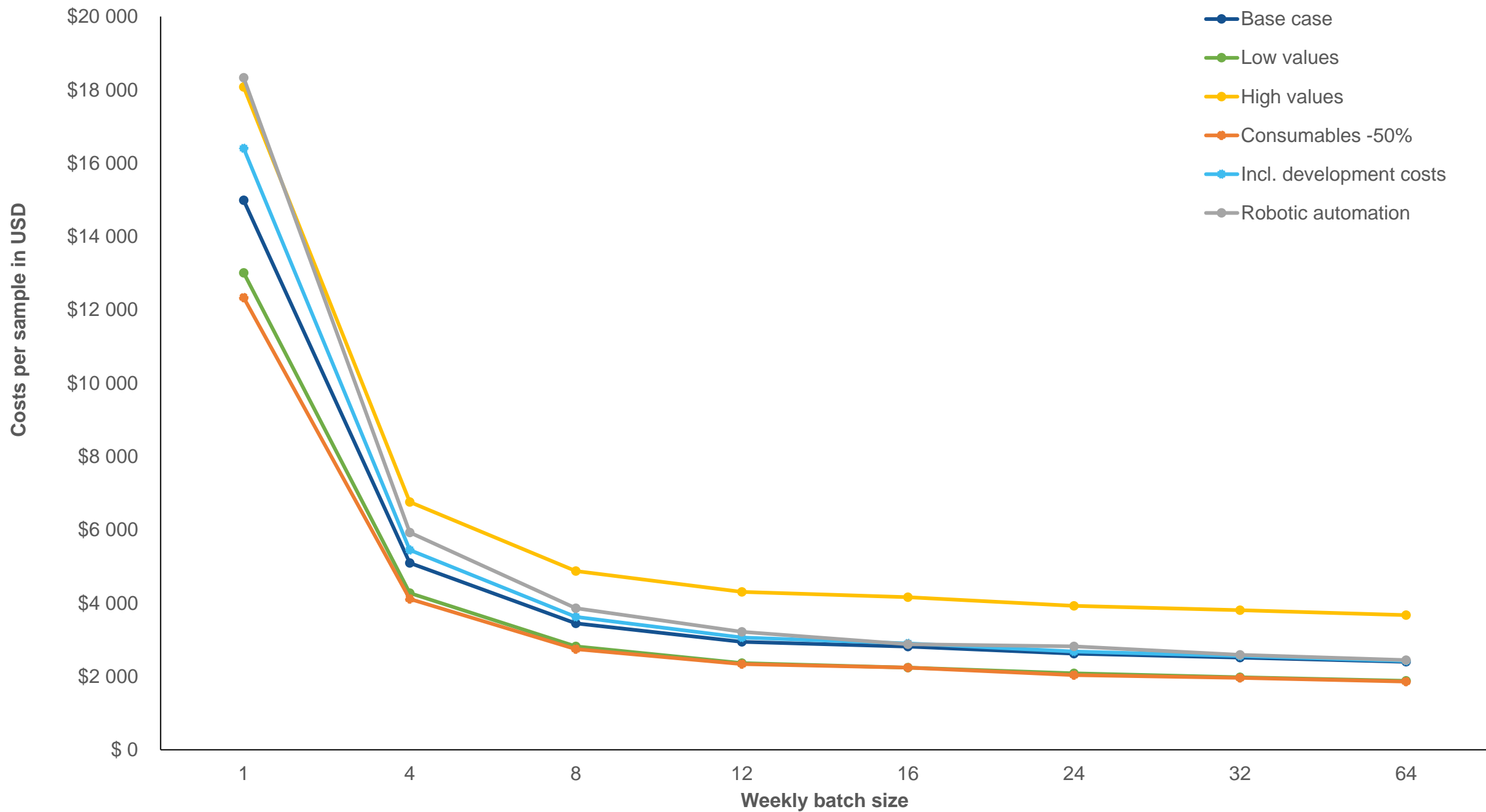

Supplement: Supplemental Figure 1 — Costs per sample in scenario analyses along different batch sizes. This line chart displays the costs per sample along different batch sizes for the base case calculation using best estimates, low value and high value estimates for working time and equipment lifetime, and for the calculations in scenario analysis. [file mmc2.pdf]
