# Supplementary material for: Microcosting Study of Genomic Profiling for Precision Cancer Medicine: Application from the National Infrastructure for Precision Diagnostics in Norway
Source: J Mol Diagn. 2025 Jul 23;27(10):945–53. doi: 10.1016/j.jmoldx.2025.06.006 (PMC12597535; doi:10.1016/j.jmoldx.2025.06.006)
Supplement: Supplemental Table S1 [file mmc3.docx]

**Supplemental Table 1.** Costs per sample with manual library preparation for a weekly sample size of 12 using best estimates, low value and high value estimates for working times, equipment lifetime and data storage volume. This table displays cost per sample considering that for working times, equipment lifetime and data storage volume, a range was indicated for some of these measurement input parameters. Costs for consumables and software are constant, since the number of consumables and the number of software licenses was indicated as a single value, not as a range. Overhead costs differ since they are calculated as a 20% mark-up on total costs per sample.

| Step / Cost category |  | Consumables | Personnel | Equipment | Software and storage | Overhead 20% | Total costs per sample | % of total costs |
| --- | --- | --- | --- | --- | --- | --- | --- | --- |
| 1 Analysis request and sampling | Best estimate | $0 | $150 | $0 | $0 | $30 | $179 | 6 % |
|  | Low value | $0 | $81 | $0 | $0 | $16 | $98 | 4 % |
|  | High value | $0 | $427 | $0 | $0 | $85 | $512 | 12 % |
| 2 Sample registration and processing | Best estimate | $21 | $107 | $12 | $0 | $28 | $168 | 6 % |
|  | Low value | $21 | $65 | $11 | $0 | $19 | $116 | 5 % |
|  | High value | $21 | $191 | $13 | $0 | $45 | $270 | 6 % |
| 3 DNA/RNA extraction | Best estimate | $33 | $94 | $26 | $0 | $31 | $183 | 6 % |
|  | Low value | $33 | $65 | $23 | $0 | $24 | $144 | 6 % |
|  | High value | $33 | $119 | $31 | $0 | $37 | $219 | 5 % |
| 4 Library preparation | Best estimate | $688 | $101 | $56 | $0 | $169 | $1 014 | 34 % |
|  | Low value | $688 | $79 | $44 | $0 | $162 | $974 | 41 % |
|  | High value | $688 | $119 | $73 | $0 | $176 | $1 056 | 25 % |
| 5 Sequencing | Best estimate | $273 | $31 | $279 | $25 | $122 | $729 | 25 % |
|  | Low value | $273 | $24 | $240 | $25 | $112 | $674 | 28 % |
|  | High value | $273 | $39 | $331 | $25 | $134 | $801 | 19 % |
| 6 Data Analysis | Best estimate | $0 | $48 | $0 | $0 | $10 | $57 | 2 % |
|  | Low value | $0 | $13 | $0 | $0 | $3 | $16 | 1 % |
|  | High value | $0 | $225 | $0 | $0 | $45 | $270 | 6 % |
| 7 Data interpretation | Best estimate | $0 | $292 | $0 | $3 | $59 | $353 | 12 % |
|  | Low value | $0 | $162 | $0 | $3 | $33 | $198 | 8 % |
|  | High value | $0 | $525 | $0 | $3 | $106 | $633 | 15 % |
| 8 Molecular tumor board and reporting | Best estimate | $0 | $207 | $1 | $0 | $41 | $249 | 8 % |
|  | Low value | $0 | $115 | $1 | $0 | $23 | $139 | 6 % |
|  | High value | $0 | $443 | $1 | $0 | $89 | $532 | 12 % |
| 9 Storage | Best estimate | $0 | $0 | $0 | $9 | $2 | $10 | 0 % |
|  | Low value | $0 | $0 | $0 | $7 | $1 | $8 | 0 % |
|  | High value | $0 | $0 | $0 | $10 | $2 | $12 | 0 % |
| Total costs | Best estimate | $1 015 | $1 029 | $374 | $36 | $491 | $2 944 | 100 % |
|  | Low value | $1 015 | $604 | $318 | $35 | $394 | $2 366 | 100 % |
|  | High value | $1 015 | $2 088 | $448 | $38 | $718 | $4 307 | 100 % |
| Percentage of total costs | Best estimate | 34 % | 35 % | 13 % | 1 % | 17 % | 100 % |  |
|  | Low value | 43 % | 26 % | 13 % | 1 % | 17 % | 100 % |  |
|  | High value | 24 % | 48 % | 10 % | 1 % | 17 % | 100 % |  |
